# Enhanced Perception of User Intention by Combining EEG and Gaze-Tracking for Brain-Computer Interfaces (BCIs)

**DOI:** 10.3390/s130303454

**Published:** 2013-03-13

**Authors:** Jong-Suk Choi, Jae Won Bang, Kang Ryoung Park, Mincheol Whang

**Affiliations:** 1 Division of Electronics and Electrical Engineering, Dongguk University, 26 Pil-dong 3-ga, Jung-gu, Seoul 100-715, Korea; E-Mails: jjongssuk@dgu.edu (J.-S.C.); bangjw@dgu.edu (J.W.B.); parkgr@dgu.edu (K.R.P.); 2 Division of Digital Media Technology, Sangmyung University, 7 Hongji-dong, Jongno-gu, Seoul 110-743, Korea; E-Mail: whang@smu.ac.kr

**Keywords:** EEG signal, gaze-tracking, two wearable devices, speller UI system

## Abstract

Speller UI systems tend to be less accurate because of individual variation and the noise of EEG signals. Therefore, we propose a new method to combine the EEG signals and gaze-tracking. This research is novel in the following four aspects. First, two wearable devices are combined to simultaneously measure both the EEG signal and the gaze position. Second, the speller UI system usually has a 6 × 6 matrix of alphanumeric characters, which has disadvantage in that the number of characters is limited to 36. Thus, a 12 × 12 matrix that includes 144 characters is used. Third, in order to reduce the highlighting time of each of the 12 × 12 rows and columns, only the three rows and three columns (which are determined on the basis of the 3 × 3 area centered on the user's gaze position) are highlighted. Fourth, by analyzing the P300 EEG signal that is obtained only when each of the 3 × 3 rows and columns is highlighted, the accuracy of selecting the correct character is enhanced. The experimental results showed that the accuracy of proposed method was higher than the other methods.

## 1. Introduction

An electroencephalogram (EEG) measures electrical signals from a human scalp. The EEG signals are used in many fields. For example, the EEG signals are used to diagnose diseases such as epilepsy, dementia, and attention deficit hyperactivity disorder (ADHD) [1–3]. Games involving attention and meditation that are based on EEG signals are also used for treatments [4]. There are two techniques that can be used to measure brainwave signals: invasive and non-invasive techniques [3]. Invasive techniques obtain signals from chips (electrode grid) inserted into the head. The advantage of this technique is that it can obtain accurate brainwave data. However, it has a great disadvantage in that it requires an operation to insert the equipment into the head [3]. As an alternative, the non-invasive technique of using electrodes attached to the scalp has been proposed. The advantage of this method is that it conveniently measures the EEG signals from worn or attached electrodes. However, the disadvantage is the presence of noise and individual variations in the EEG signals, which prevents the accurate perception of the user's intention through the EEG signals [3,5]. Brain-computer interfaces (BCIs) based on EEG signals are interfaces for controlling a computer through EEG signals instead of conventional devices such as a mouse and a keyboard [3]. Event-related potential (ERP) P300 is the most widely used for the BCI method [6]. P300 responds to a specific stimulus and it is a positive component that occurs between 200 and 500 ms after the stimulus [7]. Thus, it can be used to perceive a user's intention in a conventional speller user interface (UI) system. However, unintended EEG signals such as noise—which is caused by movements such as eye blinking and head movement—can cause the selection of the wrong character or word in a speller UI. And there are many individual variations in the EEG signals, even for ERP P300. In order to solve these problems, we newly propose a method to perceive a user's intention by combining gaze-tracking and EEG signals.

Gaze-tracking can be used to determine the user's region of intention on the basis of the eye movement [8]. There are two types of devices used to measure a user's gaze position: wearable and non-wearable devices. Gaze-tracking can be applied in many fields. Automotive safety systems improve driving safety by detecting the driver's gaze position [9]. Other applications include sports science, neuro-marketing, and human-computer interfaces [10–12]. The accuracy of wearable gaze-tracking system is generally better than that of a non-wearable system, and the wearable system can be easily combined with a wearable EEG measurement device. So, we combine a wearable wireless device for measuring the EEG signal and a wearable universal serial bus (USB) camera-based gaze-tracking device in order to measure both the EEG signal and the gaze position. The gaze position was determined on the basis of the center of the pupil and four specular reflections generated by the four near-infrared (NIR) illuminators on the four corners of the monitor. By analyzing the EEG signals on the basis of the area defined by the gaze position, the accuracy of perceiving the user's intention was enhanced in the speller UI system. In general, a speller UI system has a 6 × 6 matrix of alphanumeric characters, which has a disadvantage in that the number of characters is limited to a maximum of 36. In order to solve this problem, a 12 × 12 matrix that includes 144 characters is newly adopted in this research, and the error in perceiving the user's intention based on the EEG signals was reduced by using gaze-tracking. The rest of this paper is organized as follows. Section 2 presents the proposed device and methods. Section 3 presents the experimental results and analysis. Finally, Section 4 shows the conclusions.

## 2. Proposed Device and Methods

### 2.1. Proposed Device for EEG Measurement and Gaze-Tracking

**Figure 1.**
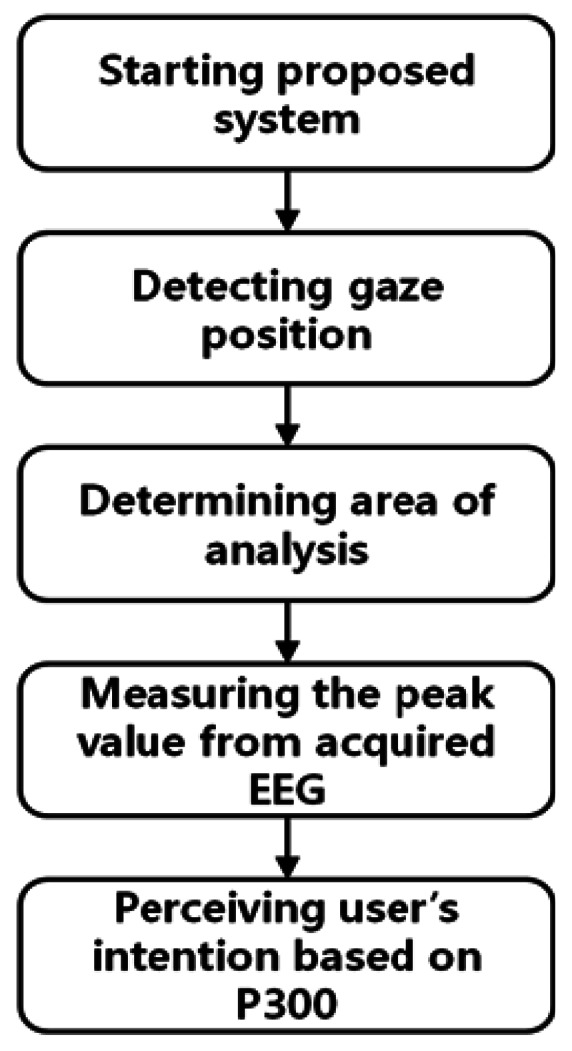
Flow chart of the proposed method.

Figure 1 shows a flowchart of the proposed method. After the proposed system is started, the user's gaze position is measured (see details in Section 2.2).

The analysis area for the EEG signal is determined on the basis of the user's gaze position. This is accomplished by checking whether peak values in the P300 EEG signal only exist when the characters of the analysis area are highlighted; this can reduce the error caused by the EEG noise and individual variations in the EEG signal. The method for analyzing the EEG signal based on P300 has been widely employed [6] (see details in Section 2.3). In this manner, the user's intention was perceived to select a specific character with reduced error.

**Figure 2.**
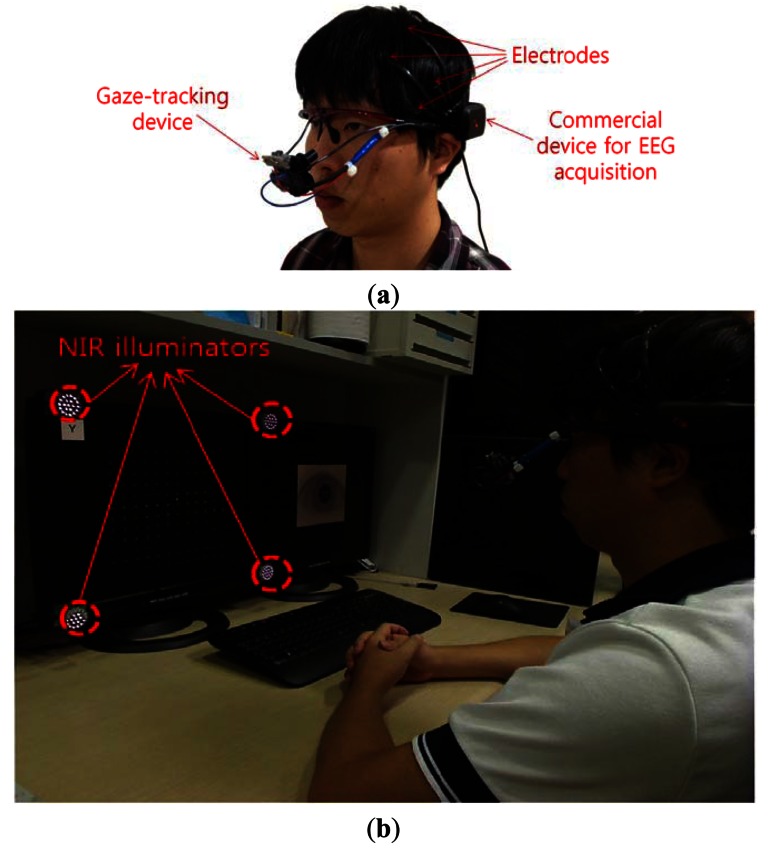
The proposed device and experimental setting. (**a**) The gaze-tracking device and a commercial device for acquiring the EEG signals. (**b**) An example of using the speller UI system by combining the analysis of the EEG signals and gaze-tracking.

**Figure 3.**
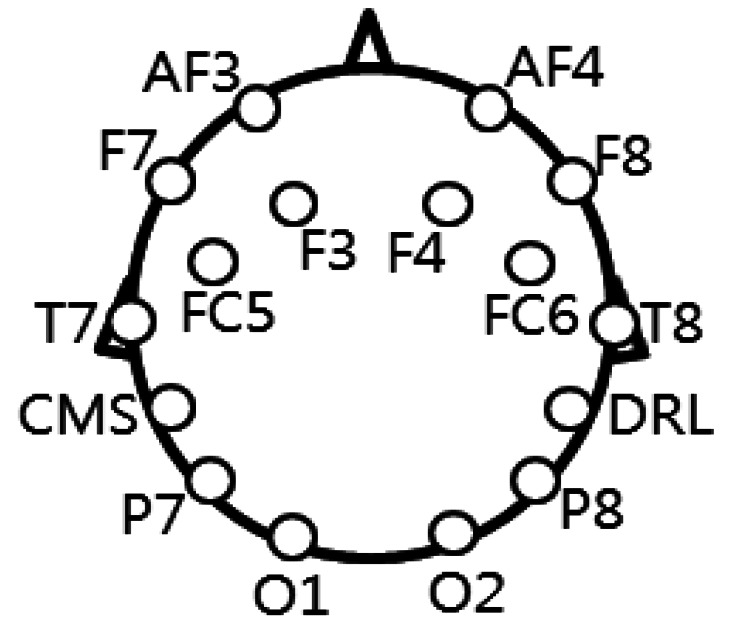
The locations of 16 electrodes.

Figure 2(a) shows the gaze-tracking device [13,14]. It uses a commercial USB web camera (Logitech C600 web camera [15]) with a zoom lens in order to acquire a larger eye image. It is a wearable device that is equipped on an eyeglasses frame with a flexible wire. As shown in Figure 2(a), a commercial headset-type device (Emotiv EPOC neuroheadset [16]) is also used to acquire the EEG signals [3]. It consists of 16 electrodes, and 2 electrodes are used as the reference point (CMS and DRL of Figure 3).

Figure 3 shows the locations of the 16 electrodes [3,17]. Although the electrode positions are roughly based on the international 10–20 system [3,17], the electrode positions (Fz, Cz, and Pz) on the entire middle line and others (P3, P4, PO7, and PO8) are not included in our device for the EEG measurement, as shown in Figure 3. The accuracy of the EEG measurement can be reduced by not using the mentioned electrode positions (Fz, Cz, Pz, P3, P4, PO7, and PO8). In order to include these electrode positions, we should use a more elaborate device for the EEG measurement. The objective of our research is to enhance the accuracy of selecting the correct character in the speller UI system with a low-cost EEG measurement device through combination with a low-cost gaze-tracking technology. Thus, if we use the more elaborate device, the accuracy of our method can be enhanced because the accuracy of using only the EEG signals based on the Emotiv EPOC device is 62.25%, as shown in Table 2.

As shown in Figure 2(b), four (custom-made) NIR illuminators are attached to the four corners of the monitor to produce four corneal specular reflections on the eye that represent the four monitor corners [13]. Twenty-one NIR light-emitting diodes (LEDs) are included in each NIR illuminator. The wavelength of the NIR illuminators is 850 nm, by which dazzling to the user's eye is minimized, and the EEG measurement of the user is consequently not affected. In general, higher contrast between the pupil and the iris areas can be obtained by using NIR light whose wavelength is longer than approximately 800 nm as compared to light whose wavelength is shorter than about 800 nm [13]. In Figure 2(b), the left-hand-side monitor indicates the speller UI system for analyzing the EEG signals, and the results of gaze-tracking are shown on the right-hand monitor.

### 2.2. Gaze-Tracking Method

The gaze-tracking algorithm operates as follows [13,14]. To find the pupil center in the captured eye image, circular edge detection (CED) is used to determine the approximate pupil position, *i.e.*, where the difference in gray levels between two nearby circular templates is maximized [13,14]. A detailed explanation of the pupil detection by CED follows. The operator of the CED is shown in Equation (1) [18]:

(1) $$\underset{(x_{0},y_{0}),r}{\arg\max}(\frac{\partial }{\partial r}\int_{0}^{2\pi }\frac{I(x,y)}{2\pi r}ds)$$

where *r* is the radius of the pupil area. Coordinates (*x_0_*, *y_0_*) are of the center position of the pupil region, and *I*(*x*, *y*) is the gray value at position (*x*, *y*). Parameters (*x_0_*, *y_0_*) and *r* (which are obtained at the moment when the calculated value of the integro-differential operation of Equation (1) is maximized) are determined as the center position and radius of the pupil area, respectively [18].

However, the pupil shape is an ellipse that is close to a circle and is distorted in the captured image. Thus, the accurate pupil position is difficult to find using only the CED algorithm. Therefore, an additional procedure is needed to accurately detect the pupil position, which is as follows [13,14]. Local binarization in the defined area (based on the detected pupil position by the CED) is performed. Morphological operations and calculation of the geometric center of the pupil region are then performed [13,14].

Four NIR illuminators [attached at the four corners of the monitor, as shown in Figure 2(b) ] generate four specular reflections in the captured eye image. The four reflections in the eye represent each corner of the monitor. These specular reflections are detected in the search area (defined on the basis of the center of the pupil) by using binarization, component labeling, and size filtering [13]. The four specular reflection positions are mapped into the four corners of the monitor by calculating the geometric transformational matrix [13]. Consequently, the gaze position on the monitor is obtained on the basis of the geometric transform and the detected pupil center. Angle kappa is the difference between the pupillary and visual axes. It is compensated for through user-dependent calibration (the user looks at the monitor center at initial stage) [13].

### 2.3. Proposed Method of Combining the Analysis of EEG Signal and Gaze-Tracking

The ERP is the brain electrical activity that is associated over a period of time with a presented stimulus with specific information [19]. In general, ERP experiments use visual and acoustic stimuli [20]. For ERP experiments, the negative peak component can be observed approximately 100–200 ms after the stimulus is presented, which are named the N100 and N200 peaks [21–23]. N400 is a negative peak component that appears 300–500 ms after the stimulus is presented [23,24]. The late positive component (LPC) is a positive peak component that appears 500–800 ms after the stimulus [23]. The method for analyzing the EEG signal based on P300 has been widely used [6]. For P300, it is reported that a positive peak component appears 200–500 ms after the stimulus is presented [7,24]. The oddball paradigm, which is a typical method used for the P300 speller UI system, is an experimental method that changes the amount of information transferred by manipulating the frequency of the stimulus [25,26].

A P300 speller UI system is used in this research. In general, a speller UI system has a 6 × 6 matrix of alphanumeric characters [27], which has a disadvantage in that the number of characters is limited to a maximum of 36. In order to solve this problem, the 12 × 12 matrix that includes 144 characters is newly employed in this research, and the error in the measurement of the P300 EEG signals is reduced by using gaze-tracking as follows.

**Figure 4.**
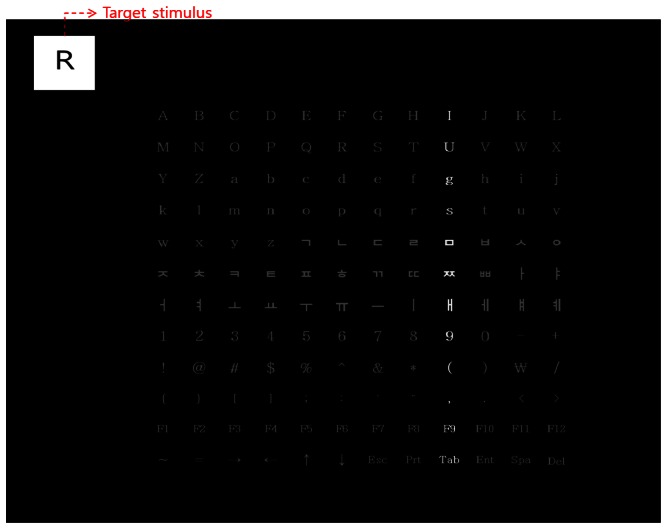
Proposed speller UI system of 12 × 12 matrix.

As shown in Figure 4, while characters are randomly shown in the target stimulus window of the upper-left position in the speller UI, the user gazes at the corresponding character in the 12 × 12 matrix (which includes the English alphabets and Korean characters, as well as numbers, special characters, and symbols, shown in Table 1). The analysis area for the P300 EEG signal is determined on the basis of the user's gaze position. This is accomplished by checking whether peak values in the P300 EEG signal only exist when the row or column including the character of the analysis area is highlighted; this can reduce the error caused by EEG noise and individual variations in the EEG signals. In this manner, the user's intention was perceived to select a specific character with reduced error.

## 3. Experimental Results

The experiments were performed on a desktop computer equipped with a 2.33-GHz CPU [Intel (R) Core (TM) 2 Quad CPU Q8200] and 4 GB of RAM. The proposed algorithm was implemented by using the C++ language with the OpenCV 2.1 library and Microsoft Visual Studio 2008. A total of 10 subjects participated in the experiment. Each subject underwent the training twice and testing 40 times for each condition by using only the EEG signals, gaze-tracking, and both the EEG signals and gaze-tracking (proposed method). As shown in Figure 4, the target stimulus was located in the upper-left part of the monitor. A specific character was randomly shown in the target stimulus window.

In the first trial of the training stage, a random character (a target stimulus of Figure 4) is shown to a person while the row or column is randomly highlighted 20 times. During this trial, the EEG signals of the person are measured when the person is looking at the same character (among 12 × 12 characters of Figure 4) as the shown one (as target stimulus). Each person conducts this trial twice for the training. In the testing stage, each person conducts the same trial 40 times. That is, 40 characters are randomly shown (as target stimulus) to one person in the testing stage. As explained, in one experiment of training or testing, each row and column of the speller UI system was randomly highlighted 20 times. Thus, each character was intensified 40 times. The inter-stimulus interval (ISI) was determined to be 125 ms (including a highlight duration of 100 ms) on the basis of the previous research [26]. For the analysis of the EEG signals, the average values of the data from all the 14 electrodes of Figure 3 are used, which can reduce the noise of the EEG signals and enhance the reliability of the analysis of the EEG signals [17].

**Figure 5.**
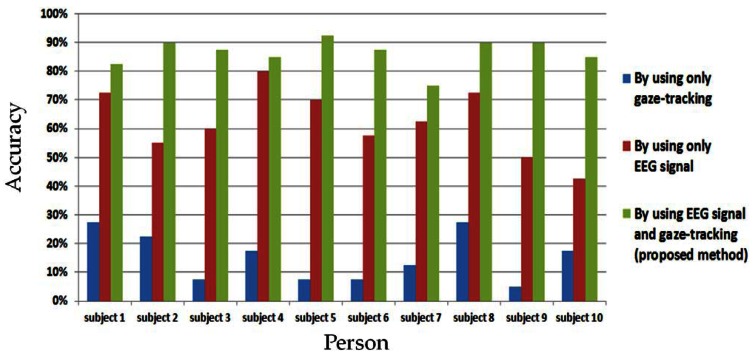
Comparisons of accuracies of three methods: checking EEG signals in the 12 × 12 matrix area without gaze position (by using only EEG signals), checking EEG signals in the 3 × 3 matrix area defined by gaze position (proposed method), and checking in the entire 12 × 12 matrix area with the gaze position (by using only gaze-tracking).

The proposed speller UI system had a 12 × 12 matrix that included the English alphabets, Korean characters, numbers, special characters, and symbols. The 12 × 12 matrix (including the interval between each character) was designed by considering a gaze error of approximately 1.12° for our system [13]. On the basis of the gaze error of 1.12° and the Z distance between the user's eye and the monitor (almost 70 cm), the horizontal or vertical gaze error in the monitor was calculated to be approximately 1.37 cm (tan 1.12° × 70 cm). Thus, the maximum resolution for selection by gaze-tracking was 1.37 cm in both the horizontal and the vertical directions; any object closer than 1.37 cm could not be discriminated simply with the help of gaze-tracking. In this study, each character (12 × 12 matrix) of Figure 4 is positioned closer than the maximum resolution of gaze-tracking, and the consequent gaze-tracking error increases with this 12 × 12 matrix, as shown in Figure 5 and Table 2. Thus, in order to solve this problem and allow the subjects to select objects placed closer than 1.37 cm, a speller UI is used by combining the gaze-tracking and the analysis of EEG signals in this research. That is, the error in gaze-tracking can be compensated for by the proposed method because the EEG signals in the rows or columns (including the 3 × 3 matrix area centered on the calculated gaze position) can be further analyzed in the proposed method. On the basis of the user's gaze position and gaze error, a 3 × 3 matrix was defined in the 12 × 12 matrix. For example, if the user's calculated gaze position is at character “S” of Figure 4, the surrounding 3 × 3 matrix area (“F,” “G,” “H,” “R,” “S,” “T,” “d,” “e,” “f”) is defined, and only three rows and three columns (including the characters of the 3 × 3 area centered on the gaze position) are randomly highlighted. Then, the presence of the maximum peak values in the P300 EEG signal is checked only when these three rows and columns are highlighted; this can reduce the error caused by the EEG noise and individual variations in the EEG signals. In this manner, the user's intention is detected to select a specific character with reduced error.

As the first experiment, the comparative accuracies of the three methods (by using only gaze-tracking, only EEG signals, and using both the EEG signals and gaze-tracking (the proposed method)) are shown in Figure 5 and Table 2. When only the gaze-tracking method of Figure 5 is used, the rows and columns are not highlighted with a shown character because the EEG measurement is not performed, and the gaze-tracking accuracy should not be affected by the highlighting. In this experiment, each participant cannot correct the error, and a case of selecting the incorrect character is counted in the error rate of Figure 5.

As shown in Figure 5 and Table 2, the average accuracy (86.5%) when checking for the EEG signals in only the 3 × 3 matrix area defined by user's gaze position (proposed method) is significantly higher than that (62.25%) when the entire 12 × 12 matrix area is checked (by using only the EEG signals). In addition, the average accuracy of the proposed method is much higher than that (15.25%) using only gaze-tracking. This means that any kind of specific character can be correctly selected with an accuracy of 86.5% by the proposed method in the speller UI systems. Although the gaze error of our system is approximately 1.12° [13], because the objects (characters of Figure 4) are closer than the maximum resolution (1.37 cm) of gaze detection, the accuracy of selecting any kind of specific character is 15.25% using the gaze-tracking method. However, this error in gaze-tracking can be compensated for by the proposed method because the EEG signals in the rows or columns (including the 3 × 3 matrix area centered on the calculated gaze position) are further analyzed in the proposed method. Although the accuracy of the proposed method is higher than those of the other methods, there still exists an error rate of 13.5(100 − 86.5)%. This can be reduced by further study of the removal of the EEG noise (caused by movements such as eye blinking or head movements) or individual variations in the EEG signals. In addition, by utilizing a highly expensive device for the acquisition of EEG signals, the accuracy can be enhanced.

**Figure 6.**
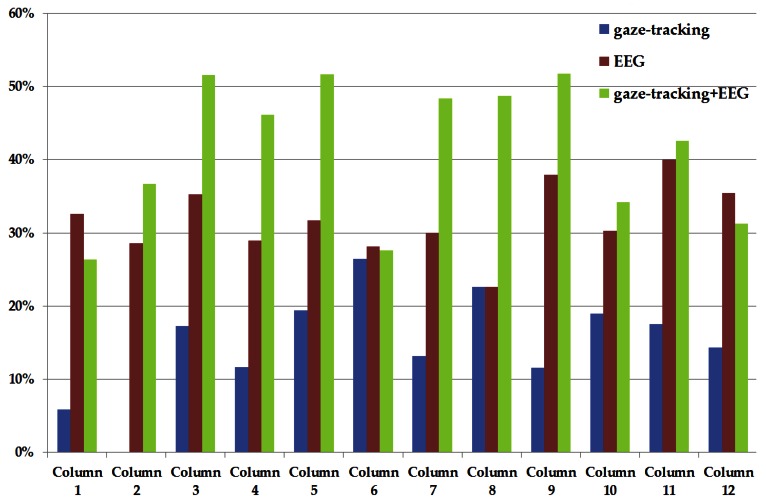
Comparisons of horizontal accuracies.

**Figure 7.**
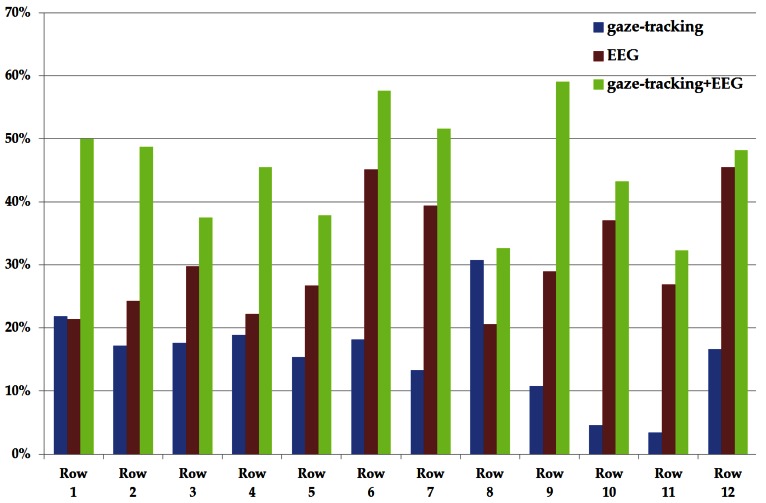
Comparisons of vertical accuracies.

Tables 3, 4 and 5 with Figures 6 and 7 show the horizontal (column) and vertical (row) accuracies of each method (by using only gaze-tracking, by using only EEG signals, and by using the proposed method, respectively). That is, they show the accuracies of selecting the characters in terms of each row or column of the 12 × 12 matrix of Figure 4. For example, the accuracies of the 1st row and 3rd column of Table 3 are 21.88% and 17.24%, respectively. As shown in Tables 3, 4 and 5 with Figures 6 and 7, the accuracy of the proposed method is higher than other two methods in terms of horizontal and vertical accuracies.

In Table 3 and Figure 6, the accuracy using only gaze-tracking is 0% in case of the 2nd column. The reason is as follows. As explained in Section 2.2 and Figure 10, the final gaze position is calculated on the basis of the detected pupil center and corneal specular reflection positions. When a user gazes at the 2nd column, the user's eye is rotated much than that in case of gazing at the center position (the 6th or 7th column). So, the corneal specular reflections are positioned in the white sclera (instead of iris area) as an elongated elliptical shape (instead of a circular shape), and the consequent detection error of the corneal specular reflections increases, which reduces the final gaze detection accuracy. However, this error in gaze-tracking can be compensated for by the proposed method because the EEG signals in the rows or columns (including the 3 × 3 matrix area centered on the calculated gaze position) are further analyzed in the proposed method. So, the accuracy (36.67%) of the 2nd column of the proposed method (as shown in Table 5) is higher than that by using only gaze-tracking.

**Figure 8.**
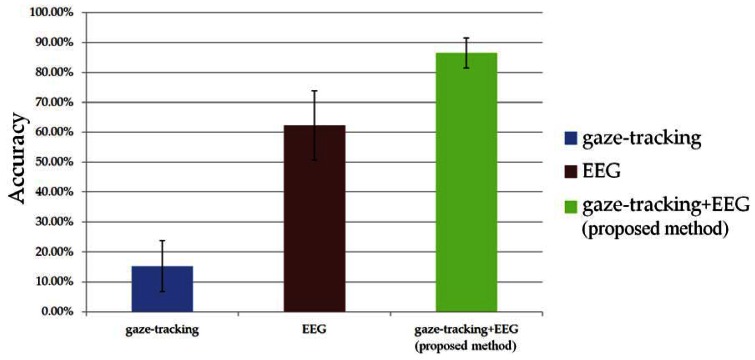
Comparisons of the average accuracies with three methods.

Figure 8 shows the average accuracies (with standard deviations) of the three methods (by using only gaze-tracking, by using only the EEG signal, by using the EEG signal and gaze-tracking (the proposed method)). On the basis of the results shown in Figure 8 and the standard deviations of each accuracy of Table 2, we performed a statistical analysis using an independent two-sample T-test [28].

The two-sample T-test has been widely used as a hypothesis test. If the calculated p value is less than the threshold based on the given confidence level, the average difference between two samples is regarded as significant [28]. The experimental results showed that the difference between the accuracies of gaze-tracking and the EEG signals was significant at a confidence level of 99% (p (9.53 × 10^−9^) < 0.01). The difference between the accuracies of the EEG signals and the proposed method was also significant at a confidence level of 99% (p (5.53 × 10^−5^) < 0.01). The difference between the accuracies of gaze-tracking and the proposed method was also significant at a confidence level of 99% (p (4.86 × 10^−13^) < 0.01). From these results, we can confirm that the accuracy of the proposed method is higher than that of the other methods at a confidence level of 99%.

**Figure 9.**
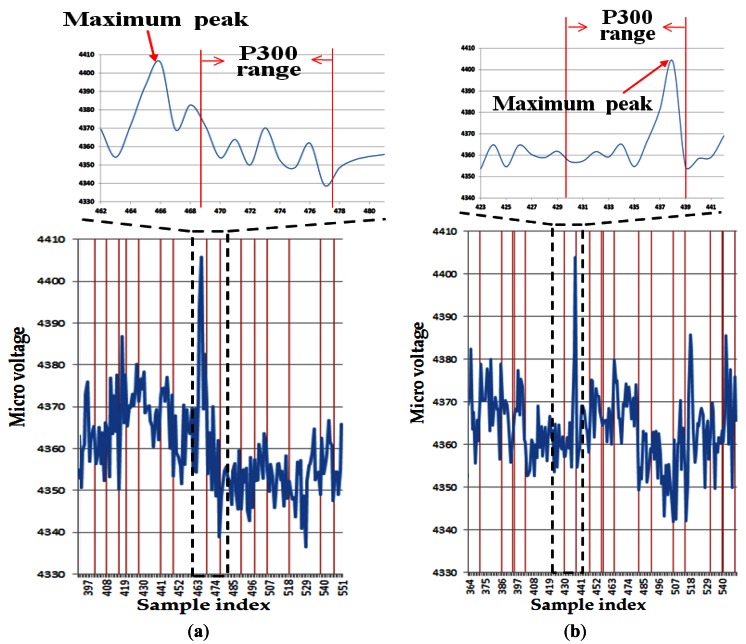
Examples of correct or incorrect detection of EEG signal. (**a**) The case of incorrect detection of the EEG signal because the maximum peak of the EEG signal does not belong to the P300 range (analysis in the entire area of the 12 × 12 matrix by only using the EEG signal). (**b**) The case of correct detection of the EEG signal because the maximum peak of the EEG signal belongs to the P300 range (analysis in the 3 × 3 matrix using the proposed method).

Figure 9 shows examples of the EEG signals acquired from the experiments with and without the proposed method. The horizontal and vertical axes show the sample index (time index) of EEG signal and micro-voltage level of the EEG signal, respectively. Figure 9(a) shows that the maximum peak does not belong to the P300 range for the analysis of the EEG signal. Thus, in this case, an error in selecting the character in the speller UI system occurs. However, Figure 9(b) shows that the maximum peak exists in the P300 range, and the character in the speller UI system can be successfully selected as a consequence. Detailed explanations of Figure 9 follow.

The EEG signal is usually checked only in the range of 200–500 ms after stimulus in the P300 scheme [7]. In the case of Figure 9(a) (where the entire area of the 12 × 12 matrix is analyzed by using only the EEG signal), the range of P300 is approximately 468 ∼ 478 in terms of the sample index. However, the maximum peak of the EEG signal appears at the index of approximately 466, which does not belong to the range of P300. Consequently, the maximum peak of the EEG signal cannot be detected in our algorithm, and this case represents the error (the error of selecting the character in the speller UI system occurs) when measuring the accuracy in Figure 5. In the case of Figure 9(b) (where the area of the 3 × 3 matrix is analyzed by the proposed method), the range of P300 is approximately 429 ∼ 439 in terms of the sample index. And the maximum peak of the EEG signal appears at the index of approximately 438, which belongs to the range of P300. Thus, the maximum peak of the EEG signal can be successfully detected in our algorithm, and this case represents the correct detection case (the character in the speller UI system can be successfully selected) when measuring the accuracy in Figure 5.

As the second experiment, we compared the proposed method with other methods in terms of the processing time. As explained at the beginning of Section 3, a total of 10 subjects participated in the experiment. Each subject underwent two trials for training and 40 trials for testing of each condition (by using only the EEG signal, by using only gaze-tracking, and by using the EEG signal and gaze-tracking, which is the proposed method). Table 6 shows the average processing time of one trial for testing. As shown in Table 6, the processing speed of the proposed method is faster than that of the method using only the EEG signal. The reason why the processing speed of the proposed method is faster than that of the method using only the EEG signal is that only a 3 × 3 matrix area is analyzed in the proposed method instead of a 12 × 12 region.

**Figure 10.**
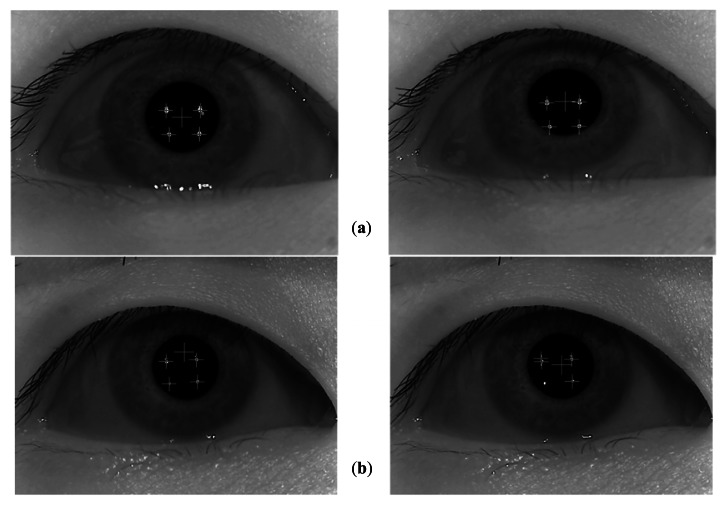
Examples of correct and incorrect detections of pupil center and corneal specular reflection positions. (**a**) The cases of correct detection. (**b**) The cases of incorrect detection.

Figure 10 shows the examples of correct and incorrect detections of the pupil center and corneal specular reflection positions. As explained in Section 2.2, because the final gaze position is calculated on the basis of the detected pupil center and corneal specular reflection positions, the gaze detection error of Figure 10(b) increases. In the case of the left-hand-side image of Figure 10(b), the pupil center is incorrectly detected, whereas the corneal specular reflections are correctly detected. In the case of the right-hand-side image of Figure 10(b), the lower-left corneal specular reflection is incorrectly detected, whereas the pupil center is correctly detected. Thus, in these cases, the gaze error becomes larger.

**Figure 11.**
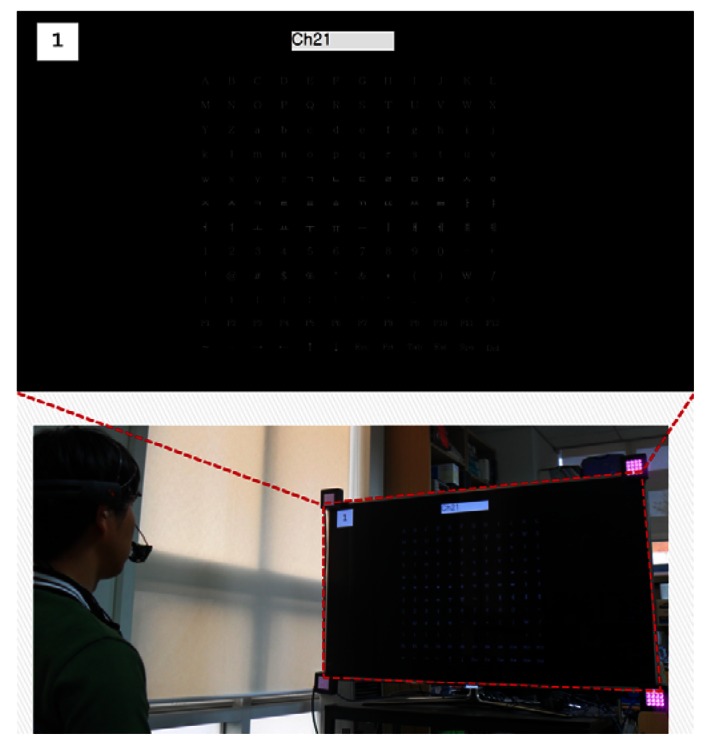
Example of the channel controller of smart TV using the proposed method.

**Figure 12.**
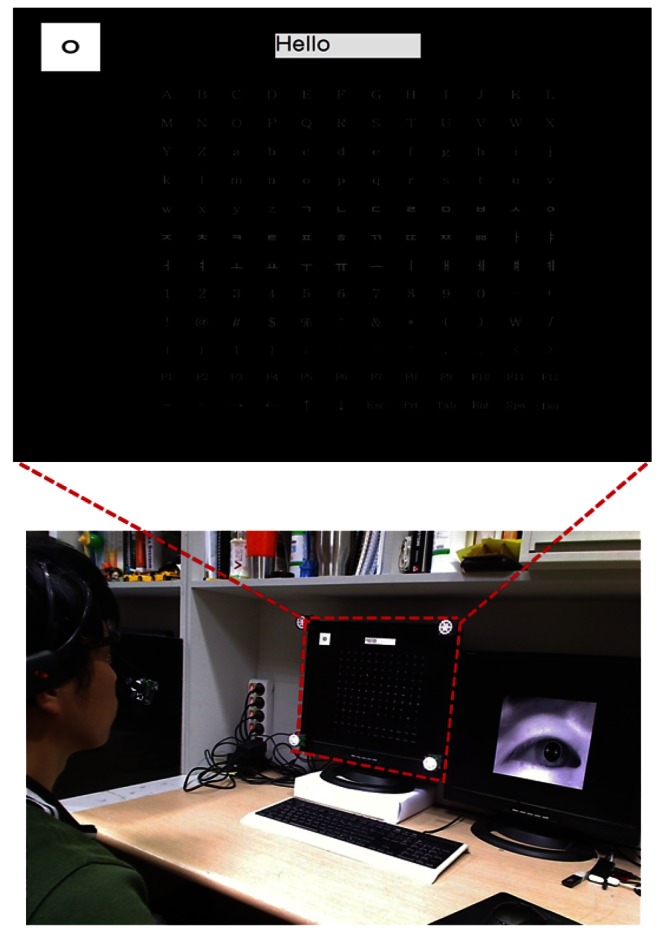
Example of the text typing system of a desktop computer using the proposed method.

As shown in Figures 11 and 12, we adopted the proposed system for the channel controller of a smart TV and a text typing system of a desktop computer. Figure 11 shows an example where a user can successfully type “Ch21” for controlling the channel on a smart TV. The Z distance between the smart TV and user is approximately 170 cm. The screen size of the smart TV is 60 inches. Figure 12 shows an example in which a user can input the text of “Hello” on a desktop computer by using the proposed method. This kind of text typing system can be used in various computer applications such as email, a web browser, and a login system. The Z distance between the monitor and the user is approximately 70 cm, and the monitor size is 19 inches. From Figures 11 and 12, we confirm that the proposed method can be used as a user interface system in various applications.

## 4. Conclusions

A new method of perceiving a user's intention in a speller UI based on ERP P300 is proposed that combines gaze-tracking and EEG analysis. In order to prevent incorrect character selection and improve the EEG analysis accuracy, the area for EEG analysis is reduced from the entire 12 × 12 matrix to a 3 × 3 matrix through gaze-tracking. The peak EEG signal is checked on the basis of P300 only after the row or column based on the 3 × 3 matrix area is highlighted. Experimental results showed that the proposed method has higher accuracy than the method that only uses the EEG signal or gaze-tracking. In future work, we would enhance the accuracy of our method by changing the number of stimuli and considering various analysis methods of the EEG signals in various applications.

## Figures and Tables

**Table 1.** Descriptions of the numbers of targets and specific target letters.

The number of targets (the number of rows and columns)		144 (12 × 12)
Specific target letters		A B C D E F G H I J K L M N O P Q R S T U V W X Y Z a b c d e f g h i j k l m n o p q r s t u v w x y z ㄱ ㄴ ㄷ ㄹ ㅁ ㅂ ㅅ ㅇ ㅈ ㅊ ㅋ ㅌ ㅍ ㅎ ㄲ ㄸ ㅉ ㅃ ㅏ ㅑ ㅓ ㅕ ㅗ ㅛ ㅜ ㅠ ㅡ ㅣ ㅐ ㅔ ㅒ ㅖ 1 2 3 4 5 6 7 8 9 0 ― + ! @ # $ % ^ & * ( ) W̶ / { } [ ] ; : ' " , . < > F1 F2 F3 F4 F5 F6 F7 F8 F9 F10 F11 F12 ∼ = → ← ↑ ↓Esc Prt Tab Ent Spa Del
Kinds of letters	Capital letter (26)	A B C D E F G H I J K L M N O P Q R S T U V W X Y Z
	Small letter (26)	a b c d e f g h i j k l m n o p q r s t u v w x y z
	Korean letter (32)	ㄱ ㄴ ㄷ ㄹ ㅁ ㅂ ㅅ ㅇ ㅈ ㅊ ㅋ ㅌ ㅍ ㅎ ㄲ ㄸ ㅉ ㅃ ㅏ ㅑ ㅓ ㅕ ㅗ ㅛ ㅜ ㅠ ㅡ ㅣ ㅐ ㅔ ㅒ ㅖ
	Number (10)	1 2 3 4 5 6 7 8 9 0
	Special character (32)	― + ! @ # $ % ^ & * ( ) W̶ / { } [ ] ; : ' " , . < > ∼ = → ← ↑ ↓
	Function symbol (18)	F1 F2 F3 F4 F5 F6 F7 F8 F9 F10 F11 F12 Esc Prt Tab Ent Spa Del

**Table 2.** Comparisons of the accuracies of three methods of Figure 5 (unit: %).

	**By Using only Gaze-Tracking**	**By Using only EEG Signal**	**By Using EEG Signal and Gaze-Tracking (Proposed Method)**
Subject 1	27.5	72.5	82.5
Subject 2	22.5	55	90
Subject 3	7.5	60	87.5
Subject 4	17.5	80	85
Subject 5	7.5	70	92.5
Subject 6	7.5	57.5	87.5
Subject 7	12.5	62.5	75
Subject 8	27.5	72.5	90
Subject 9	5	50	90
Subject 10	17.5	42.5	85
Average (standard deviation)	15.25 (0.085350584)	62.25 (0.11574037)	86.5 (0.05027701)

**Table 3.** Horizontal and vertical accuracies using only gaze-tracking (unit: %).

	5.88	0.00	17.24	11.63	19.44	26.47	13.16	22.58	11.54	18.92	17.50	14.29
21.88	A	B	C	D	E	F	G	H	I	J	K	L
17.14	M	N	O	P	Q	R	S	T	U	V	W	X
17.65	Y	Z	a	b	c	d	e	f	g	h	i	j
18.92	k	l	m	n	o	p	q	r	s	t	u	v
15.38	w	x	y	z	ㄱ	ㄴ	ㄷ	ㄹ	ㅁ	ㅂ	ㅅ	ㅇ
18.18	ㅈ	ㅊ	ㅋ	ㅌ	ㅍ	ㅎ	ㄲ	ㄸ	ㅉ	ㅃ	ㅏ	ㅑ
13.33	ㅓ	ㅕ	ㅗ	ㅛ	ㅜ	ㅠ	ㅡ	ㅣ	ㅐ	ㅔ	ㅒ	ㅖ
30.77	1	2	3	4	5	6	7	8	9	0	―	+
10.81	!	@	#	$	%	^	&	*	(	)	\	/
4.55	{	}	[	]	;	:	'	"	,	.	<	>
3.45	F1	F2	F3	F4	F5	F6	F7	F8	F9	F10	F11	F12
16.67	∼	=	→	←	↑	↓	Esc	Prt	Tab	Ent	Spa	Del

**Table 4.** Horizontal and vertical accuracies using only EEG signals (unit: %).

	32.56	28.57	35.29	28.95	31.71	28.13	30.00	22.58	37.93	30.30	40.00	35.48
21.43	A	B	C	D	E	F	G	H	I	J	K	L
24.32	M	N	O	P	Q	R	S	T	U	V	W	X
29.79	Y	Z	a	b	c	d	e	f	g	h	i	j
22.22	k	l	m	n	o	p	q	r	s	t	u	v
26.67	w	x	y	z	ㄱ	ㄴ	ㄷ	ㄹ	ㅁ	ㅂ	ㅅ	ㅇ
45.16	ㅈ	ㅊ	ㅋ	ㅌ	ㅍ	ㅎ	ㄲ	ㄸ	ㅉ	ㅃ	ㅏ	ㅑ
39.39	ㅓ	ㅕ	ㅗ	ㅛ	ㅜ	ㅠ	ㅡ	ㅣ	ㅐ	ㅔ	ㅒ	ㅖ
20.59	1	2	3	4	5	6	7	8	9	0	―	+
28.95	!	@	#	$	%	^	&	*	(	)	\	/
37.04	{	}	[	]	;	:	'	"	,	.	<	>
26.92	F1	F2	F3	F4	F5	F6	F7	F8	F9	F10	F11	F12
45.45	∼	=	→	←	↑	↓	Esc	Prt	Tab	Ent	Spa	Del

**Table 5.** Horizontal and vertical accuracies using the proposed method (unit: %).

	26.32	36.67	51.52	46.15	51.61	27.59	48.39	48.72	51.72	34.15	42.55	31.25
50.00	A	B	C	D	E	F	G	H	I	J	K	L
48.72	M	N	O	P	Q	R	S	T	U	V	W	X
37.50	Y	Z	a	b	c	d	e	f	g	h	i	j
45.45	k	l	m	n	o	p	q	r	s	t	u	v
37.84	w	x	y	z	ㄱ	ㄴ	ㄷ	ㄹ	ㅁ	ㅂ	ㅅ	ㅇ
57.58	ㅈ	ㅊ	ㅋ	ㅌ	ㅍ	ㅎ	ㄲ	ㄸ	ㅉ	ㅃ	ㅏ	ㅑ
51.61	ㅓ	ㅕ	ㅗ	ㅛ	ㅜ	ㅠ	ㅡ	ㅣ	ㅐ	ㅔ	ㅒ	ㅖ
32.61	1	2	3	4	5	6	7	8	9	0	―	+
59.09	!	@	#	$	%	^	&	*	(	)	W̶	/
43.24	{	}	[	]	;	:	'	"	,	.	<	>
32.26	F1	F2	F3	F4	F5	F6	F7	F8	F9	F10	F11	F12
48.15	∼	=	→	←	↑	↓	Esc	Prt	Tab	Ent	Spa	Del

**Table 6.** Comparisons of average processing times of various methods (unit: s).

**By using only EEG signal**	**By using EEG signal and gaze-tracking (proposed method)**	**By using only gaze-tracking**
13.01	11.74	8.51
